# Liposuction in Gynecomastia: An Assessment of the Suction-assisted Arthroscopic Shaver Versus Open Disc Excision Techniques

**DOI:** 10.7759/cureus.5897

**Published:** 2019-10-12

**Authors:** Aqsa Akhtar, Farhan Eitezaz, Mamoon Rashid, Ibrahim Khan, Saleem A Malik

**Affiliations:** 1 Plastic Surgery, Shifa International Hospital, Islamabad, PAK

**Keywords:** gynecomastia, liposuction, arthroscopic shaver, open disc excision

## Abstract

Introduction

Gynecomastia is a common problem of the male breasts, which imposes a great psychological burden on patients. It is mostly bilateral and frequently asymmetrical. Surgical management of gynecomastia has undergone significant changes over the past few decades. Currently, the predominant mode of treatment includes liposuction of the fibro-fatty tissue either alone or in combination with the removal of the glandular tissue by the open excision technique or arthroscopic shaver. This study aims to compare both techniques in terms of hematoma formation, nipple necrosis, reoperation, contour irregularities, acceptability of scarring, asymmetry, and patient satisfaction.

Methods

The study has been conducted at Shifa International Hospital, Islamabad, from May 2018 to September 2019. Sixty patients were included in the study. All the patients had bilateral gynecomastia and Simon’s Grade II-A or II-B. The study sample was divided into two equal groups. Group A underwent liposuction combined with open disc excision while Group B underwent liposuction coupled with disc excision via suction-assisted arthroscopic shaver. Postoperatively, all the patients received follow-up for a minimum period of six months.

Results

In a cohort of 60 patients, the mean age was 25.76±5.38 years. There were minor differences noted in terms of hematoma formation, nipple necrosis, rates of re-operation, and contour irregularities between open disc excision and arthroscopic disc excision, respectively (p-value > 0.05). About eight patients reported asymmetry in open disc excision as compared to 10 in arthroscopic disc excision. The acceptability of scarring was reported as equal in both groups. Mean patient satisfaction was based on the visual analog scale (VAS) scale was 8.25 in both groups. No statistical difference regarding patient satisfaction was noted in both groups (p-value 0.126).

Conclusion

Our study concludes that arthroscopic shaver-assisted disc excision despite being a novel and minimally invasive technique does not hold superiority over conventional open disc excision for the management of gynecomastia. Furthermore, in a developing country like Pakistan, there is a lack of expertise with the procedure and a need for more training among plastic surgeons.

## Introduction

The word gynecomastia has ancient Greek origin - gynec (female) and mastos (breast) - and was first introduced in the second century AD by Galen [1]. It refers to the benign enlargement of male breasts, which can be due to increased ductal tissue or fat/both. It has a reported incidence of 32%-40% [2]. It is mostly bilateral and frequently asymmetrical. It occurs mostly due to an imbalance in the levels of estradiol and testosterone. Abnormally increased levels of estrogen lead to ductal hyperplasia with elongation and branching of the ducts [3].

It has been classified by Webster in 1934, on the basis of the predominant type of tissue, into the glandular, fatty, and mixed types. However, based on clinical appearance, it has been classified by Simon et al. in 1973 into three grades: Grade I: minor visible enlargement with no redundant skin; Grade II: moderate breast enlargement with no redundant skin (IIA) and some redundant skin (IIB); Grade III: gross enlargement with excess skin, mimicking female breast ptosis [4].

Gynecomastia imposes a psychological burden on the patients, making them experience depression, anxiety, low self-esteem, and social phobia. Hence, the need for intervention to restore the normal masculine looks [5]. Various techniques for surgical correction of gynecomastia have been described. Direct excision using a transareolar and periareolar incision was introduced by Webster in 1946. The use of suction-assisted lipectomy was reported in 1980. However, liposuction alone has not been sufficient to remove the fibrous glandular tissue. Prado and Castillo in 2005 reported the use of an arthroscopic shaver to address the residual glandular component [6].

This study aims to compare both techniques in terms of seroma formation, hematomas, asymmetry of breasts, scars, need for re-operation, and patient satisfaction.

## Materials and methods

A prospective cohort study was done. All patients with gynecomastia from May 2018 to September 2019 were included in the study. A total of 60 patients with bilateral gynecomastia were surgically treated at Shifa International Hospital, Islamabad. The age of the patients ranged from 17 to 38 years. All patients had bilateral gynecomastia. Simon’s Grade II-A and II-B were included in the study while Simon's Grade I was excluded.

Preoperative evaluation

In the preoperative setting, a comprehensive history was taken, followed by clinical evaluation. The secondary causes of gynecomastia such as drugs and testicular tumors were ruled out. The grade of gynecomastia was recorded and pre-surgery photographs were taken in the frontal, lateral, and oblique views. Patients were educated about the procedure and possible surgical complications were explained. Signed informed consent was taken from all the patients.

Operative technique

Under general anesthesia, the patient was placed in a supine position with both arms abducted at 90 degrees. A tumescent solution was prepared (1-liter normal saline + 20 ml 2% lidocaine + 1 ml adrenaline 1:1000) and infiltrated in the surgical field. The amount of infiltrate on each side was approximately 450-700 ml. A stab incision was made bilaterally, along with the level on the inframammary fold, laterally. Suction-assisted liposuction was performed using a 3 mm cannula. Aspiration of all the fatty tissue was done. Glandular tissue was treated using disc excision in half of the patients and suction via an arthroscopic shaver in the other group.

Group 1

Following liposuction, a peri-areolar incision was made in the lower half of the nipple-areolar complex. Retraction was done in the vertical direction using a double hook skin retractor. Under direct visualization, the glandular tissue was dissected out and removed. Wound closure was done using absorbable Vicryl 3-0 and 4-0 sutures and cutaneous closure was done using monofilament Prolene 4-0 subcuticular sutures.

Group 2

After liposuction, a power and suction-assisted arthroscopic shaver was inserted through the same stab incision and the glandular tissue was approached. The tissue was excised through the constant oscillating motion of the cannulas that were moved back and forth until desired results were achieved. Special care was taken to avoid undue thinning of the areola. After the treatment of glandular tissue, a single Prolene 5-0 mattress suture was taken to close the stab incision. Sterile compression dressing was done.

Post-operative care and follow-up

The patient was monitored closely overnight for any bleeding or hematoma formation and discharged the following day. The patient was advised to continue wearing the compression garment for a period of four weeks. All patients were advised the first follow-up in the clinic on the fifth postoperative day and then the tenth postoperative day, where sutures were removed. Following this, regular activities and exercise were resumed at two weeks. Further follow-up for any residual deformity was done every week for the first four weeks and then in the third and sixth months.

## Results

A total of 60 patients were included in the studies which were divided into two groups. Each group had 30 patients. Group A underwent liposuction and open disc excision, whereas in Group B, liposuction along with suction-assisted arthroscopic shaver disc excision was done. The mean age of the patients was 25.76±5.38 years. In Group A, 14 patients had Grade II-A and 16 patients had Grade II-B while in Group B, 12 patients had Grade II-A and 18 had Grade II-B disease.

The complication rates in both techniques were comparable (Table 1).

**Table 1:** Comparison of arthroscopic disc excision with open disc excision and statistical analysis of the study

	Open Disc Excision	Arthroscopic Disc Excision	Odds Ratio	Confidence Interval		P-value
				Upper	Lower	
Nipple necrosis	0	0	0	0	0	N/A
Hematoma	6.6% (n=2)	10% (n=3)	0.643	4.15	0.1	0.640
Contour irregularities	6.6 % (n=2)	6.6 % (n=2)	1.0	7.60	0.13	1.0
Asymmetry	26.6% (n=8)	33.3% (n=10)	1.375	4.17	0.45	0.573
Re-operation	0	6.6 % (n=2)	1.071	1.17	0.97	0.150
Acceptable scarring	93.3% (n=28)	93.3% (n=28)	1.0	7.60	0.13	1.0

Hematoma formation resulted in only two patients with open disc excision and three patients faced this complication with arthroscopic disc excision. Nipple necrosis was noted in none of the patients in both groups. The development of contour irregularities was the same in both groups. In the open disc excision, eight patients developed asymmetry while in arthroscopic disc excision, a total of 10 patients were seen to have this problem. The acceptability of scarring was the same in both groups. Only two patients in arthroscopic disc excision required re-operation.

There was no statistical difference among the postoperative complications in both groups (p>0.05). The mean patient satisfaction in open disc excision was 8.6 compared to 7.8 in arthroscopic disc excision group (p=0.126).

## Discussion

Gynecomastia is a common breast pathology seen among males. In the majority of cases, it is usually self-remitting. However, in those where the condition prevails, surgical management is the most effective therapy. The ideal surgical approach includes the removal of both fatty and glandular tissue [7]. Although the trend in approaches are shifting from more to less invasive techniques, they all come with the risk of complications. A few studies have reported the surgical techniques in gynecomastia. Yet, to the best of our knowledge, the data comparing open disc excision and suction-assisted arthroscopic shaver along with liposuction in the treatment of gynecomastia is limited.

According to our study, a fractionally higher number of complications was noted in those who underwent arthroscopic shaver disc excision. While in the study done by Paul et al., a higher number of complications was noted in patients who underwent liposuction and open disc excision. Postoperative hematoma developed in two out of 15 patients in the arthroscopic shaver group (13.3%) while only one out of 15 developed hematoma in the open disc excision group (6.6%). The percentages were higher than those reported by the aforementioned study, where 1.31% of the patients developed hematoma in the arthroscopic shaver group while 3.57% of patients from the open disc excision group did so. In our study, nipple necrosis was seen in none of the patients in both groups, whereas only one patient developed partial necrosis during open disc excision according to Paul et al. No difference in contour irregularities and acceptability of scarring was noted in both the groups. The percentage of asymmetry was also higher in the cases of liposuction plus arthroscopic shaver group (n=10), i.e. 33.3%, when compared to the open disc excision group (n=8), i.e. 26.6%. None of the patients demanded a re-operation in the open disc excision group while only one patient wanted a re-operation in the liposuction plus arthroscopic shaver group. When compared to the study of Paul et al., the percentage was higher in the arthroscopic shaver group (11.8%) while only 5.3% of patients demanded a re-operation in the open disc excision group. Patient satisfaction with the procedure was scored from 1 to 10 according to the visual analog scale during their follow-up visits. Better satisfaction was noted in the open disc excision group when compared with the arthroscopic shaver group (8.6/10 vs 7.8/10). The results were contrary to the study of Paul et al., where arthroscopic shaver disc excision had a higher ratio of patient satisfaction [8]. The initial use of liposuction helps demarcate the glandular tissue, facilitating its excision and fine contouring. The open technique has the advantage of better visualization of the tissue, ensuring complete excision and better control of hemostasis. The suction-assisted arthroscopic shaver uses the same site of incision as used for the initial liposuction, leaving no scarring along the nipple. This does not guarantee complete excision, and small bleeding points can later result in an increased incidence of hematoma formation postoperatively. The constant oscillating motion of the shaver, if not performed carefully, can also result in the thinning of the skin, leading to necrosis [9].

Our study, despite being one of its kind, has a few limitations. To give a verdict regarding a better technique for gynecomastia, our sample was quite small. Additionally, there was no randomization in the choice of procedure offered to the patients. Some of our outcomes were subjected to reporting bias owing to the use of nonobjective outcome inventories.

## Conclusions

Our study concludes that arthroscopic shaver-assisted disc excision, despite being a novel and minimally invasive technique, is not superior to conventional open disc excision for the management of gynecomastia. Furthermore, in a developing country, such as Pakistan, there is a lack of expertise with the procedure and there is a need for more training among plastic surgeons.
